# Nasal paraganglioma: a case report

**DOI:** 10.1016/S1808-8694(15)31317-3

**Published:** 2015-10-20

**Authors:** Mauren P. Rocha, Andréa M. Campagnolo, Vanessa S. Macedo, Fabiana B. Scarton, Henrique P. Rocha, Gabriel Kuhl

**Affiliations:** 1 Resident physicians in Otorhinolaryngology, Hospital de Clínicas de Porto Alegre; 2 Ph.D. studies under course, Faculdade Federal de Ciências Médicas de Porto Alegre; 3 Ph.D. studies under course, Medical School, Federal University of Rio Grande do Sul; 4 Supporting Professor, Department of Ophthalmology and Otorhinolaryngology, Medical School, Federal University of Rio Grande do Sul

**Keywords:** nasal paraganglioma, nasal cavity, nasal tumour

## Abstract

Paragangliomas are tumors of the autonomic nervous system, arising from paraganglionic tissue. Paragangliomas of the head and neck region are very rare. In the head and neck, the most common sites of origin of this neoplasm are the carotid body, the jugular bulb and the vagal body. Paragangliomas of the nose and paranasal sinuses are very uncommon. The authors referred one case of nasal paraganglioma in a 45-year-old male patient, who was submitted to surgical excision, and included clinical findings, diagnostic criteria, treatment, prognosis and literature review. The importance of reporting this case refers to the rare incidence of paragangliomas in the nasal cavity and paranasal sinuses.

## INTRODUCTION

Paragangliomas are neoplasias arising from paraganglionic tissue of the autonomic nervous system. Paraganglions are of neuroectodermal origin and present two types of cells: Type I, which contain catecholamine granules; Type II, which are supportive cells similar to Schwann cells surrounding type I cells^1^.

Paraganglions are broadly distributed in the human body, found in the lungs, heart, mediastinum, gastrointestinal tract, retroperitoneal region and bladder. In the head and neck, they were found in trachea, tongue, larynx, hypophysis, pineal gland and orbit. Despite these findings, the most prevalent sites of paragangliomas are the carotid body, jugular body, along glossopharyngeal nerve and its tympanic branch, and the vagus nerve, especially next to nodal ganglion^2^.

This study reports a case of nasal paraganglioma in a 45-year-old male patient due to the rare aspect of such clinical condition at this specific site.

## LITERATURE REVIEW

Head and neck paragangliomas are very rare, with an incidence of 0.0012%^3^. In general, they are symptomatic tumors, which may be clinically taken for other benign or malignant lesions^3^. Paragangliomas are not uncommonly diagnosed as glomic tumors, as they both present intense vascularization^4^, regardless of being immune and histologically distinct. Types most frequently found are the carotid (carotid body), jugular (jugular bulb) and vagal (vagal nerve)^5^.

The site of origin of nasal paragangliomas remains unknown. Many authors suggest the existence of paraganglionic tissue in the pterygopalatine fossa, based on paraganglions' close relationship with arteries and cranial nerves. However, among the cases previously reported in literature, the majority was found in the regions of ethmoidal middle concha or ethmoidal sinus^1^.

## CASE REPORT

J.C., a 45-year-old Caucasian man searched for medical care complaining of nasal obstruction for one year. He also referred chronic nasal secretion with periods of purulent rhinorrhea. No bleeding, pain or facial edema was reported.

**Figure 1:**
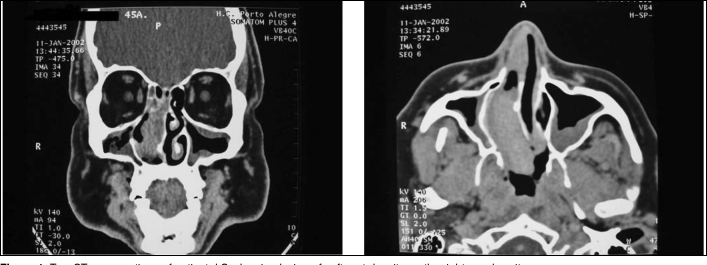
Two CT scan sections of patient J.C. showing lesion of soft part density on the right nasal cavity.

**Figure 2:**
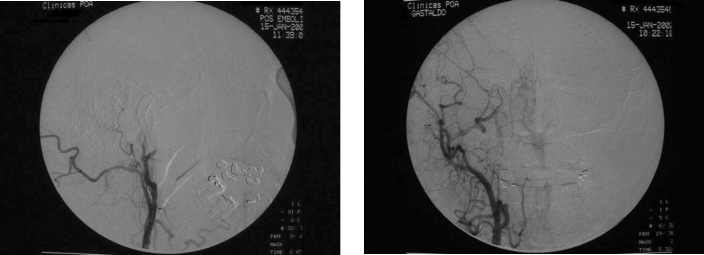
Sections of arteriography before and after maxillary artery embolization.

At otolaryngological examination, previous rhinoscopy showed a polyploid mass in the right nasal fossa. Fibroscopy revealed a lesion in the right nasal fossa obstructing the ipsilateral choanal portion. Computed tomography scan (CT) presented a lesion with dense soft tissues in the right nasal cavity (Figure 1). Incisional biopsy was performed under local anesthesia revealing a nasal paraganglioma. After previous embolization of the maxillary artery by arteriography (Figure 2), the patient was submitted to surgery with transoperative freezing process, on January 16^th^ 2002, when total excision of lesion was carried out. Both the clinical pathology and final examinations confirmed the presence of a nasal paraganglioma. No immediate or late postoperative complications were observed. After 5 months of surgery, the patient was asymptomatic and attending outpatient follow-up. So far, he has not showed evidences of relapses.

## DISCUSSION

Paragangliomas of the nose and of paranasal sinuses are very infrequent. According to a study comprising 73 cases of head and neck paragangliomas, only 3 were of nasal and paranasal types^2^. An updated literature review demonstrated only 22 cases of paragangliomas on that anatomical site^1^.

Nasal paraganglioma is a slow-growing neoplasia, with a time interval between symptoms' onset and diagnosis of 2 or more years^5^. There is a well-defined and natural tendency towards multicentricity. Many synchronic tumors are incidentally revealed during arteriography. Usually, incidence of bilaterality and multicentricity of these tumors are of 3%, going up to 26% among patients with positive family history, which corroborates family predisposition^6^.

Clinical expression is recurrent episodes of mild to profuse epistaxis, rhinorrhea, nasal obstruction and facial edema, which may be followed by blurred vision^3^, ^5^. In general, this neoplasia presents with a polyploid mass fixed to the lateral wall of the nasal fossa or on the upper region of the rhinopharyngeal roof^3^. In some cases, the paranglioma extends to the paranasal sinuses, with erosion of bone walls^5^.

**Figure 3:**
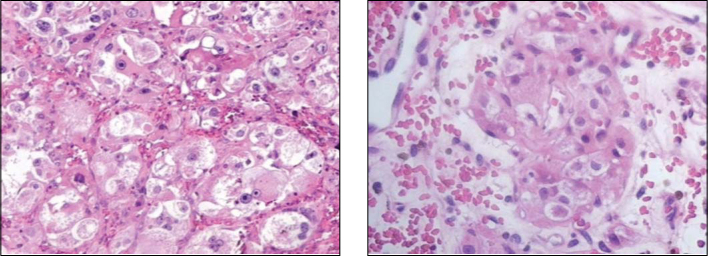
Histological sections of nasal paraganglioma showing epithelioid cells that form nests named *zellballen*, separated by a network of capillaries stained with HE with 50X and 100X.

Macroscopically, paragangliomas are hard lesions of grayish or rosy color and with encapsulated aspect^3^. Histologically, these neoplasias are peculiarly formed by epithelioid cells with round nuclei and eosinophilic cytoplasm, forming nests called *zellballen* which are separated by a rich capillary net of reticulin (Figure 3)^1^, ^5^, ^7^. Electronic microscopy revealed the presence of cytoplasmatic neurosecreting granules in these cells of the cytoplasm^1^. Some benign neoplasias present cell pleomorphism and nuclear hyperchromic aspect, including mitotic figures, which leads many authors to assume the presence of bone invasion or distant metastases in the establishment of malignancy diagnosis^1^.

Assessment and diagnosis of nasal paragangliomas must include computed tomography, nuclear magnetic resonance and arteriography with investigation of the venous phase. Arteriography also allows evaluation of collateral circulation. Tumor extension and its relation with vascular structures of the neck and skull base should be established^6^.

Use of special histological staining is important to demonstrate neurosecreting cytoplasmatic granules^3^. Histological analysis samples may be successfully obtained by means of incision biopsy of lesion, as demonstrated by Lack et al^3^. In this research study, incision biopsy provided a diagnosis of all cases of nasal (3), carotid (3), jugular (7) and orbital (1) paragangliomas, to which this technique was applied^3^.

Considering that paragangliomas present tendency towards progressive invasion of vital structures - leading to morbidity -, and also count on improved surgical techniques available, surgical excision with disease-free borders remains the treatment of choice for these neoplasias^3^. However, paragangliomas tend to locally relapse due to its nature and localization^1^. Radiotherapy for the treatment of paragangliomas is reported in the literature, but with variable results. Many authors reported an appropriate disease control with radiotherapy, although not reaching the cure. Thus, this therapeutic approach is reserved for patients without surgical indication or those with inadequate tumor excision. Chemotherapy was clearly ineffective in the treatment of paragangliomas. Embolization has been primarily used to restrict blood volume during surgery^1^, ^8^.

Paragangliomas may occur as the syndrome of multiple endocrine neoplasia (MEN) combined with medullar carcinoma of thyroid gland and, optionally, as pleochromocytoma. In such cases, endocrine evaluation and magnetic nuclear resonance of the adrenal, thoracic and neck regions are necessary for an appropriate therapeutic strategy^9^.

Malignant head and neck paragangliomas show an incidence of 4 to 19%^1^. Metastases are rare and are found in 9% of the cases^4^. Literature reports show that metastases tend to involve lymph nodes, lungs and bones^2^. It is widely accepted that malignancy potential of paragangliomas cannot be estimated by lesions' histological aspect only^2^. Not only histological findings suggestive of potential malignant behavior - such as mitotic figures, nest necrosis and vascular invasion - should be considered, but also an accurate assessment of paragangliomas presenting uncommon infiltrating growth or recurrences^4^, ^10^ should be performed. Such neoplasias should be submitted to aggressive surgical resections, once they are strongly characteristic of malignant behavior^1^. However, an aggressive intervention on these specific cases may not always lead to good results, as prognosis of patients with malignant paragangliomas are normally reserved, regardless of the lesion's site^1^.

## CLOSING REMARKS

The rare occurrence of paragangliomas of the nasal fossa has motivated our study. Once diagnosed, they should be treated surgically, considering its morbidity due to tendency towards progressive invasion of vital structures. However, even with adequate surgical approach, paragangliomas tend to present local relapse due to its nature and localization.
